# Epauletted fruit bats display exceptionally high infections with a *Hepatocystis* species complex in South Sudan

**DOI:** 10.1038/s41598-017-07093-z

**Published:** 2017-07-31

**Authors:** Juliane Schaer, Susan L. Perkins, Imran Ejotre, Megan E. Vodzak, Kai Matuschewski, DeeAnn M. Reeder

**Affiliations:** 1 Max Planck Institute for Infection Biology, Parasitology Unit, Berlin, 10117 Germany; 2 Museum für Naturkunde - Leibniz Institute for Research on Evolution and Biodiversity, Mammals Collections, Berlin, 10115 Germany; 3 American Museum of Natural History, Sackler Institute for Comparative Genomics, New York, 10024 USA; 4 Humboldt University, Institute of Biology, Berlin, 10117 Germany; 5 Bucknell University, Department of Biology, Lewisburg, 17837 USA; 6 Smithsonian Conservation Biology Institute, National Zoological Park, Washington, DC 20008 USA

## Abstract

*Hepatocystis* parasites are closely related to mammalian *Plasmodium* species, the causative agents of malaria. Despite the close phylogenetic relationship, *Hepatocystis* parasites lack the intermittent erythrocytic replication cycles, the signature and exclusive cause of malaria-related morbidity and mortality. *Hepatocystis* population expansion in the mammalian host is thought to be restricted to the pre-erythrocytic liver phase. Complete differentiation of first generation blood stages into sexual stages for subsequent vector transmission indicates alternative parasite/host co-evolution. In this study, we identified a region of exceptionally high prevalence of *Hepatocystis* infections in Old World fruit bats in South Sudan. Investigations over the course of five consecutive surveys revealed an average of 93 percent prevalence in four genera of African epauletted fruit bats. We observed a clear seasonal pattern and tolerance of high parasite loads in these bats. Phylogenetic analyses revealed several cryptic *Hepatocystis* parasite species and, in contrast to mammalian *Plasmodium* parasites, neither host specificity nor strong geographical patterns were evident. Together, our study provides evidence for Pan-African distribution and local high endemicity of a *Hepatocystis* species complex in Pteropodidae.

## Introduction

Parasites of the mammal-infecting haemosporidian parasite genus *Hepatocystis* are closely related to mammalian *Plasmodium* species^1–3^. *Hepatocystis* parasites are largely confined to arboreal mammals of the Old World tropics, and they are common in and described from Old world monkeys, fruit bats and squirrels^4^. The chevrotain (*Hyemoschus*)^5^ and *Hippopotamus amphibius* ^6^ represent less well-known hosts. The genus *Hepatocystis* currently contains up to 25 known species, but many species descriptions lack independent confirmation^7, 8^.

The first reports of *Hepatocystis* parasites in bats date back to 1926, when Rodhain described, what he thought to be a species of *Plasmodium*, *Plasmodium epomophori* from different African pteropid bats^9^. The species was later reclassified as species of *Hepatocystis* after Garnham (1950)^10^ described the asexual liver stages of *Hepatocystis epomophori* and found them to resemble *Hepatocystis kochi* from primates. All reported bat hosts to date belong to the closely related bat families Pteropodidae and Hipposideridae of the suborder Yinpterochiroptera.

The *Hepatocystis* life cycle differs from that of its closest relatives, mammalian *Plasmodium* species^8^, in several life cycle stages. In the vertebrate host, the presence of macroscopic exoerythrocytic schizonts, so-called merocysts, is the most prominent feature of *Hepatocystis*. Merocysts are formed in the liver and, similar to *Plasmodium* parasites, generate thousands of daughter cells (merozoites), but they appear to be the only replication phase in the vertebrate host. After release into the blood stream, merozoites invade erythrocytes and directly develop into sexual stages (gametocytes). Thus, contrary to *Plasmodium* species, *Hepatocystis* parasites appear to lack the asexual erythrocytic replication cycles. Accordingly, infections likely do not result in the characteristic malaria signs that are associated with this specific parasite life cycle step. It is important to note that an early report described irregular schizonts in the blood of bats^9^. However, this observation was questioned by Garnham (1953)^11^, who failed to confirm similar signatures of asexual replication in *Hepatocystis* infections and commented that these “blood-stage schizonts” were more likely fragments of the large liver merocysts.

*Hepatocystis* infections are generally described as benign, although some complications, such as anaemia and scarring of the liver, have been discussed^4^. The notion of fitness costs by *Hepatocystis* infections is strongly supported by the identification of apparent selection of resistance alleles in the promoter region of the Duffy blood group antigen/chemokine receptor *DARC* in *Hepatocystis kochi*-infected yellow baboons (*Papio cynocephalus*)^12^. Few studies of the pathogenicity of *Hepatocystis* infections in bat hosts have been conducted, however.

The arthropod vector of *Hepatocystis* also differs fundamentally from mammalian *Plasmodium* parasites, which are exclusively transmitted by anopheline mosquitoes. After many unsuccessful attempts to find the vector of *Hepatocystis* in a wide range of candidate arthropods^13, 14^, *Culicoides adersi* (Ceratopogonidae, Diptera) was eventually confirmed as the vector for the primate-infecting species *Hepatocystis kochi* ^13, 15^. Sporogony of *Hepatocystis* parasites is unusual since ookinetes encyst in the head and thorax between muscle fibres of *Culicoides* (rather than on the midgut wall, as in *Plasmodium*), but after mature oocysts rupture and release sporozoites, they migrate to the salivary glands similar to *Plasmodium* transmission^4^. Vector incrimination still awaits confirmation for the remaining *Hepatocystis* species.

In this study we present a systematic serial survey of *Hepatocystis* infections in sympatric fruit bats of the Republic of South Sudan. Bats were investigated in repeated surveys from 2010 to 2015 in both the wet and the dry season. The systematic characterization of prevalence, parasitaemia and phylogenetic relationships of *Hepatocystis* infections reveal previously unrecognized insights into this neglected mammalian haemosporidian genus.

## Results

### Prevalence

**Figure 1:**
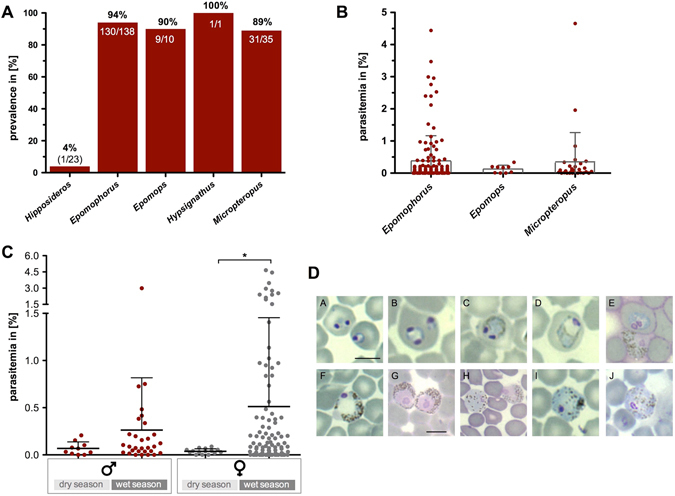
Parasitological parameters of *Hepatocystis* infections. (**A**) Prevalence of parasite infections in hipposiderid and pteropid host genera in percent (total numbers of infected individuals are listed below). (**B**) Parasitemia levels did not differ significantly between the three host genera *Epomophorus*, *Epomops* and *Micropteropus* (Chi-square = 0.945, df = 2, p = 0.624), suggesting that all three genera are similarly susceptible to *Hepatocystis* infections. Parasitemia range is given as a percentage, *i*.*e*. the number of gametocyte-infected erythrocytes in total erythrocytes and bars indicate mean parasitaemia and standard deviation. (**C**) For both sexes, parasitaemia values were higher in samples from the wet season than from the dry (Table 3), but this difference was only significant for females (females: dry season − n = 15, mean = 0.04% ± 0.03%; wet season − n = 97, mean = 0.51% ± 0.94%; t = −4.94, df = 97, p < 0.0005; males: dry season − n = 10, mean = 0.07% ± 0.07%; wet season − n = 30, mean = 0.26% ± 0.55%; t = −1.10, df = 38, p = 0.278). (**D**) Representative micrographs showing gametocyte stages of *Hepatocystis* parasites of *Epomophorus* hosts. *A–E* depict early gametocyte stages, *F*,*G* depict mature male microgametocytes, *I*,*J* depict mature female macrogametocytes and *H* depicts a macro- (left) and microgametocyte (right). Bar indicates 5 µm.

A total of 393 bats belonging to eight families and 18 chiropteran genera from five consecutive surveys in South Sudan (2010–2013, 2015) were investigated (Table S1). *Hepatocystis* parasites were verified in 172 individuals, corresponding to an overall prevalence of 44%. Individuals of four out of five examined pteropid genera, namely the epauletted fruit bats *Epomophorus*, *Epomops*, *Hypsignathus*, and *Micropteropus* harboured *Hepatocystis* parasites. The two investigated individuals of the fifth investigated pteropid bat genus *Rousettus* (*lanosus*) were *Hepatocystis-*negative. One individual out of 23 of the genus *Hipposideros* (Hipposideridae) harboured a very low infection of *Hepatocystis*. Together, infected bats were confined to the two families Hipposideridae and Pteropodidae (Table 1, Fig. 1A). Subpatent infections were recorded for 15% (n = 25) of the positive samples (detection in the PCR screening only, Table S2, Fig. S1). Notably, bats with these subpatent infections were almost exclusively sampled during the dry season.

**Table 1:** Prevalence in Pteropodidae and Hipposideridae.

Host genus	Prevalence in %					
	2010 (*Aug*, *2*.*8*.*–12*.*8*.)	2011 (*Sep/Oct*, *2*.*9*.*–12*.*10*.)	2012 (*Jul*, *18*.*7*.*–29*.*7*.)	2013 (*May/Jun*, *31*.*5*.*–1*.*6*.)	2015 (*Jan*, *1*.*1*.*–12*.*1*)	Total
	wet season	wet season	wet season	wet season	dry season	
***Hipposideros***	—	**7** (1/15)	0 (0/6)	0 (0/2)	—	**4** (1/23)
***Epomophorus***	**100** (28/28)	**93** (41/44)	**95** (19/20)	**100** (9/9)	**89** (33/37)	**94** (130/138)
***Epomops***	—	—	**90** (9/10)	—	—	**90** (9/10)
***Hypsignathus***	—	—	**100** (1/1)	—	—	**100** (1/1)
***Micropteropus***	**93** (13/14)	**67** (4/6)	**90** (9/10)	**100** (5/5)	—	**89** (31/35)
***Rousettus***	0 (0/1)	—	0 (0/1)	—		0 (0/2)

Table 1 lists prevalences of *Hepatocystis* infections in the bat host families Hipposideridae and Pteropodidae. Given are prevalences in % and total numbers of infected per total investigated individuals per host genus in the corresponding sampling year.

Among the epauletted hosts, consistently very high prevalences of *Hepatocystis* infections were verified across the different years, ranging from 89% up to 100% (Table 1, Fig. 1A). In the most representative sample size of *Epomophorus* hosts investigated across all years (n = 138), the lowest prevalence of 89% (33/37), was documented in 2015, when bats were sampled during the dry season. The mean prevalence in *Epomophorus* across all years was 94% (133/138) (Table 1). A similar high prevalence (89%) was detected for *Micropteropus* (n = 35). Moreover, most individuals of *Epomops* (9/10) and the one investigated *Hypsignathus* individual featured infections with *Hepatocystis*. Members of the families Nycteridae and Rhinolophidae featured infections with *Nycteria* parasites^16^.

### **Parasitemia**

For bats with confirmed *Hepatocystis* infections, parasitaemia values (% of infected erythrocytes) were calculated. In pteropid bat hosts, parasitaemias ranged from 0.1% to 0.4% (Table 2). As expected for cross-sectional survey, the parasitaemia range varied considerably, with minimum parasitaemia levels below the microscopic detection limit (<0.001%) and extremely high maximum parasitaemias of up to 4.7% in some individuals. Parasitemia in the single individual of *Hipposideros* was below 0.001%. Parasitemia levels did not differ significantly between the three host genera of epauletted bats that made up the bulk of our samples, *Epomophorus*, *Epomops* and *Micropteropus* (Chi-square = 0.945, df = 2, p = 0.624) (Fig. 1B), suggesting that all three genera are similarly susceptible to *Hepatocystis* infections.

**Table 2:** Parasitemia *of Hepatocystis* infections – host genera.

Bat host genus	n	M ± SD* in %	Min (in %)	Max (in %)
*Epomophorus*	114	0.42 ± 0.81	<0.001	4.44
*Epomops*	9	0.13 ± 0.12	<0.001	0.34
*Hipposideros*	1	/	<0.001	<0.001
*Hypsignathus*	1	/	0.50	0.50
*Micropteropus*	29	0.35 ± 0.91	<0.001	4.66

Table 2 Mean parasitaemia (M), Standard Deviation (SD) as well as maximum (Max) and minimum (Min) parasitaemia of *Hepatocystis* infections are given in % for each bat genus.

For the epauletted species, within each sex, there were no significant differences in parasitaemia levels between adults and juveniles (females: t = 0.216, df = 110, p = 0.829; males: t = 0.018, df = 38, p = 0.985; Table 3), thus age classes were pooled for subsequent comparisons. Overall, females had significantly higher parasitaemia levels than males (females: n = 112, mean = 0.45% ± 0.89%; males: n = 40, mean = 0.21% ± 0.49%; t = −2.053, df = 125.3, p = 0.042).

**Table 3:** Parasitemia values in different sex and age groups.

Sex	Season	Age/pregnancy	n	M ± SD* in %	Max (in %)*
**female**		***all***	**112**	**0**.**45 ± 0**.**89**	**4**.**66**
		*adult*	74	0.44 ± 0.83	4.44
		*juvenile*	38	0.47 ± 1.01	4.66
	dry season	*all*	15	0.04 ± 0.03	0.09
		*pregnant*	11	0.04 ± 0.02	0.08
		*non pregnant*	4	0.03 ± 0.04	0.09
	wet season	*all*	97	0.51 ± 0.94	4.66
		*pregnant*	5	0.11 ± 0.15	0.34
		*non pregnant*	92	0.53 ± 0.96	4.66
**male**		***all***	**40**	**0**.**21 ± 0**.**49**	**2**.**99**
		*adult*	32	0.21 ± 0.53	2.99
		*juvenile*	8	0.22 ± 0.26	0.75
	dry season	*all*	10	0.07 ± 0.07	0.21
	wet season	*all*	30	0.26 ± 0.55	2.99
**female + male**		*adult*	108	0.37 ± 0.75	4.44
**female + male**		*juvenile*	46	0.43 ± 0.93	4.66

Table 3 lists parasitaemia values of *Hepatocystis* infections sorted in different sex and age host groups. Given are mean parasitaemia (M) and Standard Deviation (SD) as well as maximum parasitaemia (Max) in %, *minimum parasitaemia for all groups <0.001%. Within each sex, there were no significant differences in parasitaemia levels between adults and juveniles (females: t = 0.216, df = 110, p = 0.829; males: t = 0.018, df = 38, p = 0.985). Overall, females had significantly higher parasitaemia levels than males (females: n = 112, mean = 0.45% ± 0.89%; males: n = 40, mean = 0.21% ± 0.49%; t = −2.053, df = 125.3, p = 0.042). For both sexes, parasitaemia was higher in samples collected in the wet season than in the dry (Fig. 1C), but this difference was only significant for females (females: dry season – n = 15, mean = 0.04% ± 0.03%; wet season – n = 97, mean = 0.51% ± 0.94%; t = −4.94, df = 97, p < 0.0005; males: dry season – n = 10, mean = 0.07% ± 0.07%; wet season – n = 30, mean = 0.26% ± 0.55%; t = −1.10, df = 38, p = 0.278). The very high levels of parasitaemia found in some females during the rainy season were not related to pregnancy. In fact, parasitaemia was lower in pregnant females during the wet season, although not significantly (pregnant females: n = 5, mean = 0.11% ± 0.15%; non pregnant females – n = 92, mean = 0.53% ± 0.96%; t = 0.976, df = 95, p = 0.332). The majority of pregnant females captured were from the dry season, when parasitaemia was universally low. Age, sex, seasonal differences and the influence of pregnancy were assessed with t-tests where values were corrected for unequal variances when necessary.

For both sexes, parasitaemia was higher in samples collected in the wet season (May-October) than in the dry (January) (Fig. 1C, Table 3), but this difference was only significant for females (females: dry season – n = 15, mean = 0.04% ± 0.03%; wet season – n = 97, mean = 0.51% ± 0.94%; t = −4.94, df = 97, p < 0.0005; males: dry season – n = 10, mean = 0.07% ± 0.07%; wet season – n = 30, mean = 0.26% ± 0.55%; t = −1.10, df = 38, p = 0.278). Highest mean parasitaemias of 0.84% ( ± 1.25) as well as highest maximum parasitaemia of 4.66% were noted in bats from 2012 that were sampled in late July in Western Equatoria State (Table 4). The lowest mean parasitaemias of 0.05% ( ± 0.05) were detected in January (2015) in the dry season, followed by samples from May, at the onset of the wet season. The very high levels of parasitaemia found in some females during the rainy season were not related to pregnancy. In fact, parasitaemia was lower in pregnant females during the wet season, although not significantly (pregnant females: n = 5, mean = 0.11% ± 0.15%; non pregnant females – n = 92, mean = 0.53% ± 0.96%; t = 0.976, df = 95, p = 0.332). The majority of pregnant females captured were from the dry season, when parasitaemia was universally low.

**Table 4:** Parasitemia *of Hepatocystis* infections across sampling seasons.

Month(s)	Year	Sampling site	n	M ± SD* in %	Max (in %)	Min (in %)
01.–12. Jan	2015	Central Equatoria State	25	0.05 ± 0.05	0.21	<0.001
31. May–01. Jun	2013	Western Equatoria State	13	0.18 ± 0.21	0.73	<0.001
18.–29. Jul	2012	Western Equatoria State	37	0.84 ± 1.25	4.66	<0.001
02.–12. Aug	2010	Central Equatoria State	35	0.24 ± 0.58	2.96	<0.001
02. Sept–12. Oct	2011	Central Equatoria State	40	0.39 ± 0.70	3.47	<0.001

Table 4 lists number of infected individuals, sampling month(s) and year, their corresponding mean parasitaemia (M) and Standard Deviation (SD) as well as maximum (Max) and minimum (Min) parasitaemia in %.

### **Morphology of parasite blood stages**

The only parasites visible in the blood of *Hepatocystis-*infected animals are gametocyte stages^4^. The morphology of early and mature gametocyte stages detected in this study largely corresponds to the description of *Hepatocystis epomophori* ^4, 9^. The youngest parasites are small ring-shaped forms with a solid nucleus and double-infections of the erythrocytes are common. The development of a vacuole in the ring forms is apparent and sometimes the nucleus splits into two or more granules (Fig. 1D A–B). The hemozoin is only visible once the vacuole starts disappearing. The pigment is fine-grained and the colour ranges from light green to dark brown (Fig. 1D C–D). Mature macro- and microgametocytes are distinguishable by the colour of the cytoplasm after Giemsa staining. Macrogametocytes (Fig. 1D H–J) appear as bright blue whereas the microgametocytes (Fig. 1D F–H) exhibit a biscuit-coloured cytoplasm and a rose-coloured area around the nucleus, which is free of pigment. In the majority of the infections seen in the current study, blood stages were limited to mature gametocytes. Approximately 8% of the infections exhibited both young and mature stages in the blood simultaneously; all were sampled during the wet season when parasitaemias were high.

### **Unusual Hepatocystis blood stages**

Three individuals of *Epomophorus* sp. (DMR630, DMR631, DMR634), sampled during the wet season, featured blood stages that differed from the preceding descriptions and instead resembled Rodhain’s^9^ notes on the early blood stages that he thought to be erythrocytic schizont stages. At the early stage, blood stages are annular or ovoid shaped and exhibit a peripheral nucleus (Fig. S2A). Additionally, we observed a separation of the nucleus in two or three parts that seemed to be isolated from each other and only connected by the parasite’s cytoplasm. These forms were sometimes difficult to distinguish from multiple infections of a single erythrocyte by several early stages (Fig. S2B–E). Mid-adult stages tend to have an amoeboid shape (Fig. S2C,D,K,L). The cytoplasm stains pale blue and contains one or more chromatic masses and can be present in dense forms and also in small compact blocks, rounded or elongated, arranged in linear or irregular clusters that give the parasite a characteristic appearance (Fig. S2A,E–O). We also observed some parasites that were apparently in the process of division (e.g. Fig. S2I). Rodhain noted multiple forms of division in “irregular schizonts” that were quite numerous in two samples and he further indicated a division into six up to twelve blocks. In conclusion, in three out of 172 infections, which displayed high parasitaemia, blood stages that display features of asexual replication were detected, indicative of an alternative, but rare, *Hepatocystis* expansion phase in host erythrocytes. It cannot, however, be ruled out that these blood stages present the onset of the process of exflagellation of microgametocytes, artificially initiated during preparation of the blood smears.

### **Phylogenetic diversity of infections and generalism among African fruit bat hosts**

**Figure 2:**
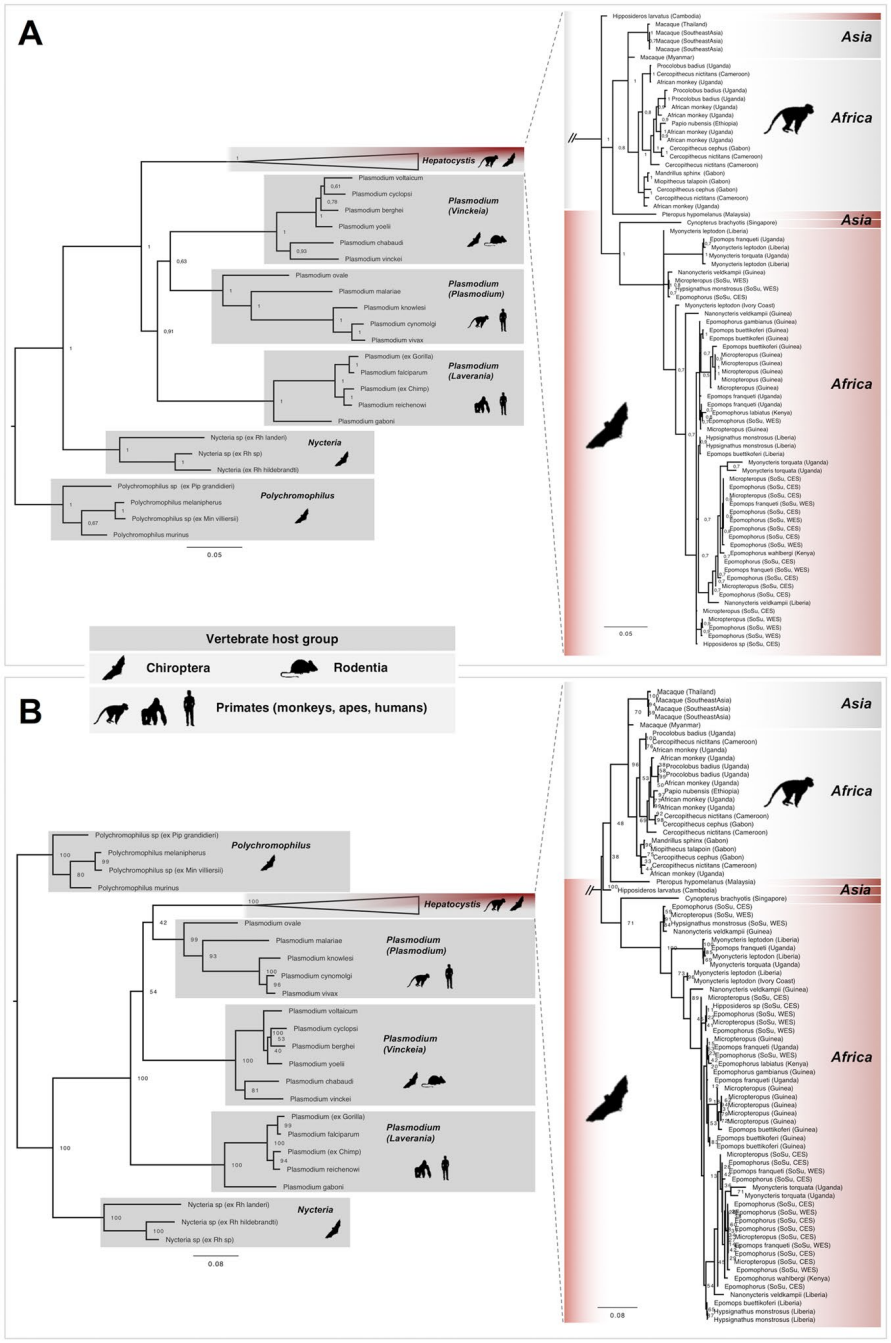
Three-genome phylogeny for *Hepatocystis* parasites in the context of the mammalian haemosporidian parasite clades. The concatenated phylogenies were obtained via analysis of four genes, the mitochondrial cytochrome *b* and cytochrome oxidase 1, the apicoplast caseinolytic protease and the nuclear elongation factor 2. The *Hepatocystis* clade is shown as collapsed clade (on the left) and this section is enlarged and uncollapsed on the right site. The *Hepatocystis* clade falls in two distinct groups, the primate *Hepatocystis* clade with the exception of two samples recovered from the bat hosts *Pteropus hypomelanus* and *Hipposideros larvatus* and an African fruit bat *Hepatocystis* clade with the exception of a sample recovered from the Asian fruit bat *Cynopterus brachyotis*, which each group as sister to the main groups respectively. (**A**) Three-genome phylogeny for *Hepatocystis* parasites recovered by Bayesian analysis. Posterior probability values are given. Placement of *Hepatocystis* parasites as sister to the mammalian *Plasmodium* clade with good support (1). (**B**) Three-genome phylogeny for *Hepatocystis* parasites recovered by maximum likelihood analysis. Bootstrap values are given. Placement of *Hepatocystis* parasites as sister to the mammalian *Plasmodium vivax/malariae* clade with low support (42).

**Figure 3:**
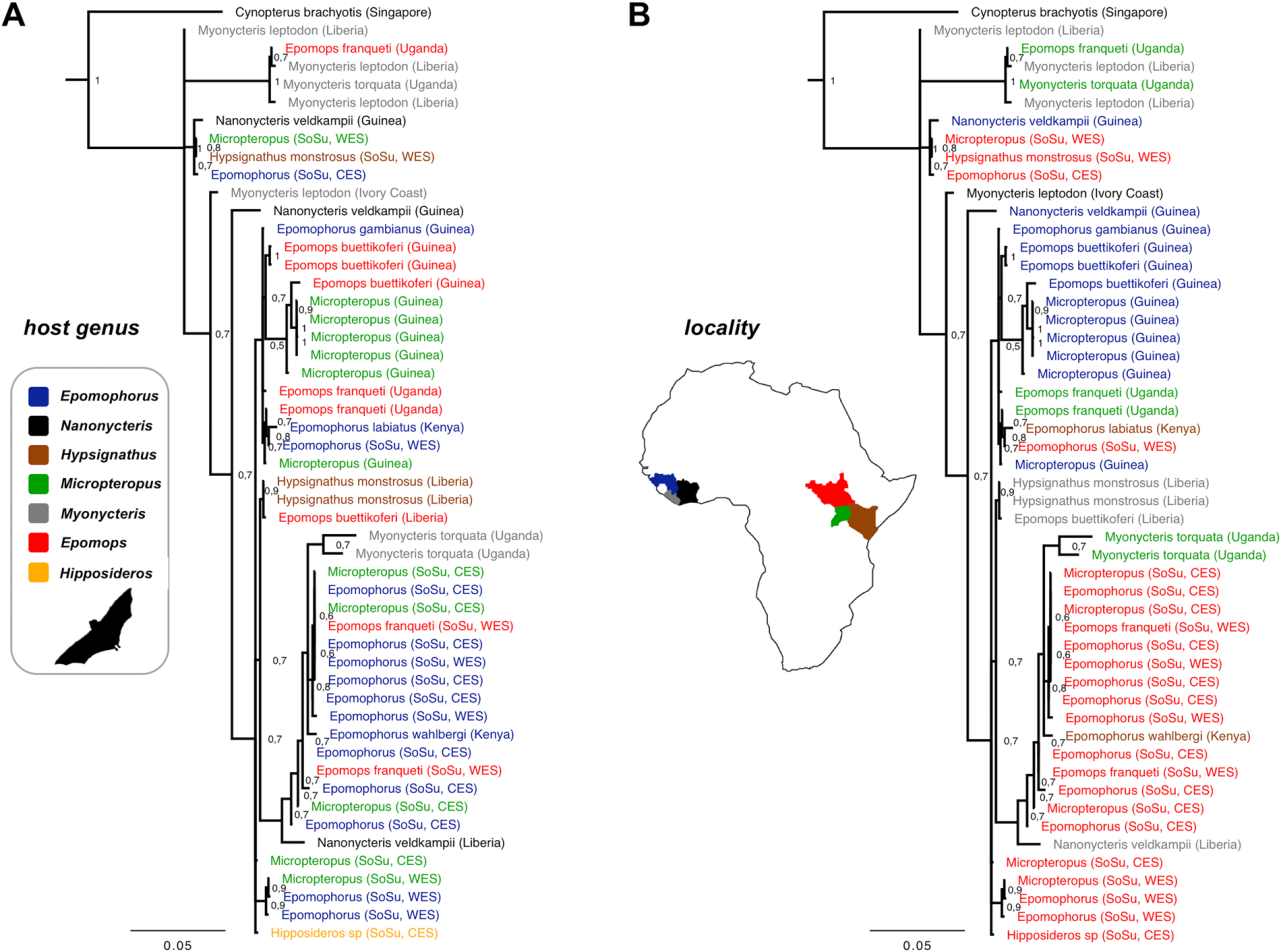
Molecular phylogeny of African bat *Hepatocystis* parasites (section of Fig. 2). (**A**) *Hepatocystis* sequences are color-coded by the seven different bat host genera (blue = *Epomophorus*, black = *Nanonycteris*, brown = *Hypsignathus*, green = *Micropteropus*, grey = *Myonycteris*, red = *Epomops*, yellow = *Hipposideros*). No strict clustering of the parasite lineages according to their associated host genus is apparent. (**B**) *Hepatocystis* sequences are color-coded by sampling localities in West Africa (blue = Guinea, grey = Liberia, black = Ivory Coast) and East Africa (red = Republic of South Sudan, green = Uganda, brown = Kenya). No definite geographical pattern is obvious as parasite sequences from West and East African sampling localities are mixed throughout the phylogenetic tree. (Map was created in Adobe Illustrator CS6 Version 16.0.0., http://www.adobe.com/de/products/illustrator.html).

Phylogenetic analyses were performed to assess the phylogenetic diversity, and possible geographic and/or host-specificity patterns of *Hepatocystis* parasites from the Republic of South Sudan. Representative infections were chosen from each host genus as well as from both sampling areas, Central Equatoria State and Western Equatoria State. In addition, published sequences of *Hepatocystis* parasites from other African (West Africa: Guinea, Liberia; East Africa: Uganda, Kenya, Mozambique) and Asian countries were included, comprising parasites from both primate and bat hosts (Supplemental Table S3; Figs 2 and 3).

Maximum likelihood and Bayesian analyses confirmed *Hepatocystis* as a monophyletic clade with high support (posterior probability of 1, bootstrap value of 100) (Fig. 2). Both analysis recovered the clades of *Plasmodium* (*Plasmodium*), *Plasmodium* (*Vinckeia*), *Plasmodium* (*Laverania*), *Nycteria* and *Polychromophilus* as monophyletic clades and relationships among the species within the clades show identical topologies (with slight differences in the *Vinckeia* clade). Bayesian analysis groups *Hepatocystis* as sister clade to the mammalian *Plasmodium* clade, which has been reported by Lutz (2016)^17^ (Fig. 2A). However, the maximum likelihood analysis resulted in a sister relationship of *Hepatocystis* with the *Plasmodium ovale/vivax* clade, as shown before^2^ (Fig. 2B).

The *Hepatocystis* clade itself is comprised of two main subclades. The first includes all parasites of primates, which forms a monophyletic clade, consisting of Asian as well as African primate hosts, with the latter presenting a monophyletic subgroup (Fig. 2, highlighted in grey). The second main group within the *Hepatocystis* clade contains all African chiropteran *Hepatocystis* parasites. Surprisingly the parasites of the Asian flying fox species *Pteropus hypomelanus*, samples in Malaysia^18^, and of *Hipposideros larvatus* from Cambodia^19^ cluster basal to the ‘primate’ *Hepatocystis* clade and not with the other chiropteran *Hepatocystis* parasites (Fig. 2). Furthermore, within the second, “African bat” clade, an Asian fruit bat (*Cynopterus brachyotis* from Singapore^3^) was host to the most basal parasite sequence (Fig. 2). The analysis indicates two transition events of *Hepatocystis* parasites from Asian bat hosts, one into (Asian and African) primates and another single introduction into African bats.

Within the African fruit bat *Hepatocystis* clade, parasite sequences do not cluster in specific clades, but represent several close related taxa or cryptic species (Fig. 3A,B). The whole clade contains parasite sequences from all six fruit bat host genera, *Epomophorus*, *Epomops*, *Micropteropus*, *Hypsignathus*, *Nanonycteris* and *Myonycteris* (highlighted with different colours in Fig. 3A), which are mixed throughout the tree. Thus, no structuring is apparent to indicate strict host specificity (Fig. 3A). Strikingly, even the parasite sequence of the second bat host family Hipposideridae does not group outside, but falls well within the clade of sequences of the African pteropid hosts.

The analysis included sequences from different countries and locations in both West Africa (Guinea, Liberia, and Ivory Coast) and Central-/East Africa (South Sudan, Uganda, Kenya), presenting considerably distant sampling areas (Fig. 3B). *Hepatocystis* from South Sudan fall in several different places across the tree, each enclosing parasites of different hosts, showing no pattern of clustering according to sampling sites within the country (Western Equatoria State (WES) and Central Equatoria State (CES)). Again, sequences of parasites of all different countries are mixed throughout the tree and the lack of structuring indicates no strict geographic patterns (Fig. 3B).

In summary, parasites from different host genera group closely together as well as do parasite lineages from very distant localities in West and East Africa, underlining that *Hepatocystis* is a generalist among the African epauletted fruit bats.

## Discussion

In the current study, we report previously unrecognized high prevalences of *Hepatocystis* infections in African epauletted fruit bats throughout different months and seasons in host individuals of both sexes and all ages. This finding could either point to chronic infections of *Hepatocystis* or to a high rate of new infections in one individual on a regular basis throughout the year. The majority of *Hepatocystis-*infected bats exhibited only mature gametocytes in the blood at the time of sampling, but some individual bats that were captured in the peak of the wet season featured early and mature gametocyte stages in the blood simultaneously. In combination with higher mean parasitaemias of bats that were captured during the wet season, these observations indicate that *Hepatocystis-*infections feature a seasonal pattern, which is consistent with earlier tentative observations^4^ and most likely correlates with the life history of the invertebrate vector. Nonetheless, the extremely high rate of infections in African epauletted fruit bats is striking and points to highly efficient transmission cycles.

The maximum parasitaemia levels in *Hepatocystis* infections of over 4% gametocyte stages inside erythrocytes would represent hyperparasitemia in human *Plasmodium* infections. Such a parasitaemia level might be tolerable in the bats since the infected red blood cells do not burst due to the lack of blood schizogony and repeated infection cycles, one critical parameter for malaria-related anaemia. However, whether high parasitaemia, which in the case of *Hepatocystis* equals high gametocytemia, impacts the oxygen-carrying capacity of the blood in these hosts and thereby, results in reduced host fitness remains to be analysed. Indeed, the first study tackling the issue of potential fitness costs of *Hepatocystis* infections revealed the evolution of a malaria resistance gene in baboons (*Papio cynocephalus*)^12^.

Parasites of the genus *Hepatocystis* have been recognized in fruit bats since 1908, first in Australian and Asian *Pteropus* species^11^ and later in several African fruit bats^20, 21^. These first notes only contained the description of gametocyte stages in the peripheral blood. Some years later, Rodhain reported the presence of schizonts in the blood of fruit bats, which lacked pigment and exhibited an irregular form with 8 to 10 blocks of chromatin, but he did not observe mature schizont stages in the blood^9^. In his review of bat malaria parasites, Manwell mentioned Rodhain’s finding and did not exclude the possibility that erythrocytic schizonts exist in *Hepatocystis* parasites^22^. In fact, he reported “segmenters” in the blood smears of the *Hepatocystis-*infected Australian flying fox *Pteropus* (*alecto*) *gouldii* ^22^. Manwell pointed out that these “segmenters” were extracellular and lacked hemozoin pigment at this stage, but stated that the finding of segmenting forms in the peripheral blood were the most remarkable results of his study^22^. The “unusual blood stages” that were documented in the current study occurred in three individuals only and should, therefore, not be dismissed, but also not be overinterpreted. Garnham (1966)^4^ argued that the blood stages, which were reported by Rodhain^9^ and Manwell^22^ represented microgametocytes that had undergone rapid development during the time interval of fixation and drying the blood smear. He reasoned that a delay in drying the material lets to the division of the nucleus of the microgametocyte prior to exflagellation. Even though all samples of the current study were prepared in the same manner, the authors cannot rule out that ambient humidity or a slight delay in preparation created conditions that might have been suitable for exflagellation to start (in three samples) and therefore would have to be considered artifacts. Certainly, future studies, including transcriptional profiling of *Hepatocystis*-infected erythrocytes and systematic organ sampling, are needed to gain a better understanding of a potential flexibility of *Hepatocystis* life cycle progression in the mammalian host. Whether the process of erythrocytic schizogony in the parasite life cycle has been gained or lost independently multiple times within haemosporidian parasites still represents one of the most important unresolved questions.

The taxonomic status and the phylogenetic placement of *Hepatocystis* among haemosporidian taxa has been highly unstable throughout the years. The first molecular studies that included *cytb-*sequences of *Hepatocystis* ^1, 2^ both recovered a paraphyletic status of the mammalian *Plasmodium* clade; with the *Hepatocystis* sequences nested within the *Plasmodium* (*Plasmodium*) and *Plasmodium* (*Vinckeia*) species. This relationship was subsequently supported by a three-genome phylogeny of haemosporidian parasites^3^. Even though the phylogenetic classification of *Hepatocystis* within the mammalian *Plasmodium* clade has been verified from several subsequent studies^23, 24^, the exact phylogenetic placement among the mammalian *Plasmodium* species still remains uncertain. In the majority of analyses *Hepatocystis* groups as sister clade to the primate *Plasmodium* clade containing *e*.*g*. *P*. *ovale* and *P*. *knowlesi* ^2, 23, 25, 26^. In other studies, however, *Hepatocystis* falls as sister to a clade comprising bat/rodent and primate *Plasmodium* species^24^, or as sister clade to a monophyletic clade that contains all mammalian *Plasmodium* species^17^. The current study could not resolve the exact placement of *Hepatocystis*, but underscores once again its close relationship with mammalian *Plasmodium* species, despite the striking differences in their respective life cycle strategies.

Species of *Hepatocystis* are described from the four mammalian host orders Cetartiodactyla, Primates, Rodentia and Chiroptera, but the majority of species has been described from the latter host group (10 out of 23 *Hepatocystis* species^8^). The main difficulty in recovering the phylogenetic relationships of *Hepatocystis* parasites is the heterogeneity of available sequences and the sampling bias within the different mammalian host groups. Until today, the majority of sequences comprise partial sequences of the *cytochrome b* gene only. Furthermore, no phylogenetic studies have been published for parasite species from artiodactyl and rodent hosts yet (except that some rodent *Hepatocystis* sequences have been published in GenBank). Considering only primate and bat *Hepatocystis* parasites, this study revealed specificity on the taxonomic level of mammalian host order, but not on host genus level, a very unusual attribute for parasites, which typically display clear signatures of close parasite/host associations and co-evolution. Different genera of bats contained *Hepatocystis* sequences that are nearly identical and only differ by a few bases. Phylogenetic studies of African epauletted fruit bats have confirmed most of the chiropteran morpho-genera and -species. However, recent studies found the genus *Epomophorus* to be paraphyletic due to the inclusion of the genus *Micropteropus*, but this remains to be confirmed by future studies^27, 28^. This, combined with the fact that *Epomophorus* and *Micropteropus* species often roost in the same trees, may explain how similar haplotypes of *Hepatocystis* are found in these two closely related bat taxa. However, similar *Hepatocystis* sequences are also found in phylogenetically more distant related genera such as *Hypsignathus* and *Myonycteris* that clearly group outside the *Micropteropus/Epomophorus* complex. Most strikingly, even the bat genus *Hipposideros*, which belongs to an entirely different bat family and does not share ecological preferences exhibits similar *Hepatocystis* sequences. Further analysis of parasites from Asian and also Australian bat hosts will be needed to explain the close relationship to the primate or the African bat *Hepatocystis* clade and to further investigate the degree of host specificity. Nonetheless, it is already apparent that a taxonomic revision of the whole *Hepatocystis*/mammalian *Plasmodium* group is required.

Infections were limited to the bat families, Pteropodidae and Hipposideridae (Suborder Yinpterochiroptera), the only two (out of 21) chiropteran families known to harbour *Hepatocystis* parasites^8^. We wish to highlight that this also applies to the chiropteran *Plasmodium* species, which again have only been described from pteropid and hipposiderid hosts. However, in contrast to *Hepatocystis*, the species of *Plasmodium* are each restricted to one distinct host species. The current study revealed several cryptic species of parasites, which, based on blood stage morphology, all belong to the species *Hepatocystis epomophori* ^4^ and lack signatures of host specificity. Similarly, our phylogenetic analysis did not recover clear strong geographical patterns of chiropteran *Hepatocystis* parasites. Some structure into East African versus West African *Hepatocystis* sequences can be observed, but in other cases, closely related parasite lineages are found on both sides of the African continent. For instance, some *Hepatocystis* sequences recovered from the bat species *Micropteropus pusillus* in Guinea are more closely related to parasites from individuals of the bat genus *Epomophorus* from South Sudan as to parasites from Guinean *Epomophorus* or South Sudanese *Micropteropus*. The low divergences that were recovered in the phylogenetic analysis indicate that these parasites are moving back and forth amongst the different host genera regularly. This broad host and geographical distribution might also imply that *Hepatocystis epomophori* can infect previously unrecognized host genera.

Four distinct *Hepatocystis* species have been formally described from African bat hosts^9, 29–31^. However, the African chiropteran *Hepatocystis* lineages appear to represent a species complex of taxa that are morphologically and phylogenetically closely related. It is tempting to speculate that *Hepatocystis* parasites were more successful in chiropteran hosts over the course of evolution than species of *Plasmodium*. This hypothesis could explain the constrictive host spectrum and high specificity of *Plasmodium* in bats on the one hand, where only two *Plasmodium* species each specific to one bat species, and apparently restricted to West Africa have been identified thus far and the wide host spectrum of at least ten host species throughout tropical Africa, but unspecific character of *Hepatocystis* parasites on the other hand. However, it should also be noted at this point that *Hepatocystis* parasites are apparently universal in epauletted fruit bats and close related taxa (comprising *Myonycteris* as well as the epauletted host genera *Micropteropus*, *Epomophorus*, *Epomops*, *Nanonycteris*, *Hypsignathus*), but no infections have been reported from the African fruit bat genera *Megaloglossus*, *Plerotes* and *Rousettus* yet, which present the most closely related bat taxa^28^. The two samples of *Rousettus* (*Stenonycteris*) *lanosus* in this study also did not harbour *Hepatocystis* parasites. Probably differences in the life history of bat species co-determine the exposure to potential invertebrate vectors and the susceptibility to *Hepatocystis* parasites, *e*.*g*. species of *Rousettus* are cave-dependent, whereas epauletted fruit bat species feature roosting sites in the foliage of trees. Further molecular and statistical analysis of *Hepatocystis* infections from different countries and hosts will be needed to test for possible host and geographical patterns in more depth.

The systematic survey of *Hepatocystis* parasites in the South Sudanese epauletted fruit bats across different years and seasons revealed consistently high parasite prevalences in a family of bats (Pteropodidae) that has been associated with diverse viruses^32^. In this context, parasite infections may affect the health of their hosts and thus might influence the response to viral infections. Pteropid bats often roost in close proximity to humans and individuals with high parasite loads might then pose a potential risk of zoonotic virus transmission. On the other hand, *Hepatocystis* infections might represent a form of commensalism with low or no impact on their vertebrate hosts and future studies are needed to determine the physiological consequences for the hosts. Despite being a close relative of mammalian *Plasmodium*, parasites of the genus *Hepatocystis* have so far been rather neglected in studies of malaria parasites. This study illustrates that *Hepatocystis* research might contribute to a better understanding of the evolution and to new insights into the biology of the whole parasite group.

## Material and Methods

### Ethics statement

All surveys were reviewed and approved by the Institutional Animal Care and Use Committee of Bucknell University (Pennsylvania, USA) and the South Sudanese Ministry for Wildlife Conservation and Tourism. All work was performed in accordance with the relevant guidelines and regulations regarding care and use of animals.

### Field sampling

Bats were sampled during five consecutive studies on mammalian biodiversity, conservation and disease ecology in southern regions of the Republic of South Sudan between 2010 and 2015^33, 34^. The surveys covered different habitat types. The region in the former Central Equatoria State on the southern border with Uganda, comprises a mix of subtropical and moist savannah, whereas the research areas further west (in the former Western Equatoria State; state names currently in flux), bordering the Democratic Republic of Congo, encompassed the tropical zone of South Sudan. Bats were captured with mist-nets and several keys were used for bat identifications^35–39^. Standard measurements were recorded for every bat to verify morphological field identification. Blood samples were collected as blood dots on filter paper or FTA cards. Thin blood smears were prepared on glass slides and fixed in 100% methanol. All bats were deposited as voucher specimens in the mammal collection of the National Museum of Natural History in Washington D.C. (NMNH) (catalogue numbers listed in Table S2) and bat species identification was confirmed by further morphological comparisons of skins, skulls, and palatal ridges. Amy T. Gilbert and her team provided a subset of pteropid bat samples collected in Kenya in 2009.

### Parasitemia

Giemsa-stained thin blood smears were examined at 1,000x with bright-field microscope and immersion oil. The mean number of erythrocytes per field was determined by counting two random fields, and the number of parasites was recorded in 20 to 30 fields, choosing fields of comparable erythrocyte density. Parasitemia was calculated as the percentage of erythrocytes infected with *Hepatocystis* parasites.

Planned comparisons were made to assess the effects of taxon, age, sex, season, and reproductive condition on parasitaemias. Due to our inability to achieve normality and homogeneity of variance in our overall dataset, a Kruskal-Wallis test was used to explore differences between the three genera of bats from which we had adequate samples. Age, sex, seasonal differences and the influence of pregnancy were assessed with t-tests where values were corrected for unequal variances when necessary. Samples from a total of 152 bats in the host genera *Epomophorus*, *Epomops* and *Micropteropus* were included in the final analysis.

### Molecular methods

The QIAGEN DNeasy extraction kit (Hilden, Germany) was used for DNA isolation from the dried blood dots on filter paper and from FTA cards (GE Healthcare). The protocol for animal tissues was performed with the minor modification of elution of the samples in 50 μl AE buffer. PCR was performed using the QIAGEN TopTaq Master Mix with 2–3 μl of genomic DNA as the template, and 1 μl of each primer (10 mM). Four genes from the three parasite genomes were targeted for subsequent phylogenetic analysis: the mitochondrial genes cytochrome *b* (*cytb*) and cytochrome oxidase 1 (*cox1*); the apicoplast caseinolytic protease gene (*clpc*); and the nuclear gene elongation factor 2 (*EF2*). Primers are listed in Table S4. All PCR products were sequenced with the amplification primers in both directions using BigDye v3.0 (Applied Biosystems) and run on an ABI-373 sequencer. Parasite sequences included 906 nucleotides (nt) of the *cytb*, 951 nt of *cox1*, 564 nt of *clpc* and 567 nt of the *ef2*-gene (Table S5).

### Screening for *Hepatocystis*

All Giemsa-stained thin blood smears were screened with the microscope for a minimum of six minutes per slide. DNA was isolated for all pteropid and hipposiderid samples and tested by PCR using the primer set Hep-F3/Hep-R3^23^, which was designed for chiropteran *Hepatocystis* (targeting a 506 bp sequence of cytochrome *b*). PCR negative samples (in the *cytb*-screen) were subsequently screened via PCR targeting the *clpc-* and/or the *ef2*-gene to confidently exclude false-negatives.

### Phylogenetic analyses

The gene sequences revealed genetically mixed infections (different haplotypes) in many samples, visible as double nucleotide peaks in the sequence electropherograms, which is also common in *Hepatocystis* infections of primates^40^. For the subsequent phylogenetic analysis, any sample with a high number of double nucleotide peaks was excluded and double peaks in the sequences that were chosen for analyses were treated as ambiguous sequences and coded as N’s (IUPAC nucleotide code for “any base”). Sequences were assembled and aligned in *Geneious* 8.1.9 using MUSCLE^41^. Corresponding sequence data from representatives of the major haemosporidian groups were included in the phylogenetic analysis (Table S3). Phylogenetic relationships were evaluated by using Bayesian inference and maximum-likelihood (ML) methods. Data were divided into partitions according to genes and *PartitionFinder v*.*2* ^42^ was used to test different DNA substitution models and partition schemes. Twelve data blocks defining first, second and third codon positions of the four protein-coding genes were determined for the concatenated alignment and best partition schemes and models were used for the phylogenetic analyses (summarized in Table S5 and Table S6). *RaxmlGUI v*.*1*.*3* ^43^ was used for the ML analysis with concatenated alignments and nodal support was evaluated using 500 thorough bootstrap pseudoreplicates^44^. Bayesian inference was conducted in *MrBayes v3*.*2*.*6* ^45, 46^ via the CIPRES Science Gateway Web Portal V3.3^47^ with two runs of four chains (three heated, one cold, temperature = 0.10) each for 20 million generations. For each independent partition a GTR + I + Γ type model was used and reversible rate matrices, partition-specific rate multipliers and stationary state frequencies had a Dirichlet prior. The α and proportion of invariant sites had uniform priors. A prior of all topologies equally likely was used for τ and the prior on branch lengths was set as unconstrained exponential (parameter 10). The first 25% of trees were discarded as burn-in. Mixing and convergence of runs, and effective sample size (ESS > 200) were assessed in the program *Tracer v1*.*6* ^48^. Runs were combined using *LogCombiner v1*.*8*.*2*, and the maximum clade credibility tree was summarized with *TreeAnnotator v*.*1*.*8*.*2*. Phylogenetic trees were visualized in *FigTree* (http://tree.bio.ed.ac.uk/software/fgtree/).

## Electronic supplementary material

Supplemental Information
