# Supplementary material for: Epauletted fruit bats display exceptionally high infections with a Hepatocystis species complex in South Sudan
Source: Sci Rep. 2017 Jul 31;7:6928. doi: 10.1038/s41598-017-07093-z (PMC5537238; doi:10.1038/s41598-017-07093-z)
Supplement: Supplementary file 1 — Supplemental Information [file 41598_2017_7093_MOESM1_ESM.pdf]

SUPPLEMENTAL MATERIAL

Epauletted fruit bats display exceptionally high infections with a *Hepatocystis* species complex in South Sudan

Juliane Schaer, Susan L. Perkins, Imran Ejotre, Megan E. Vodzak, Kai Matuschewski, DeeAnn M. Reeder

Contents:

- **Supplemental Figures S1 – S2**
- **Supplemental Tables S1 – S6**

Schaer *et al.*, Supplemental Figure S1

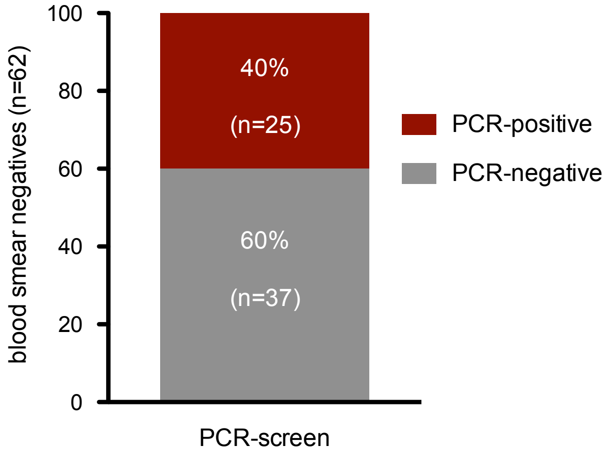

Supplemental Figure S1. PCR screening for *Hepatocystis* samples.

Samples of the two bat host families Hipposideridae and Pteropodidae were screened for haemosporidian infections via microscopy. All blood-smear negative samples were subsequently screened via PCR targeting different parasite genes and 40% (n=25) were positive for *Hepatocystis* infections. These subpatent infections were mostly recorded for the dry season.

Schaer *et al.*, Supplemental Figure S2

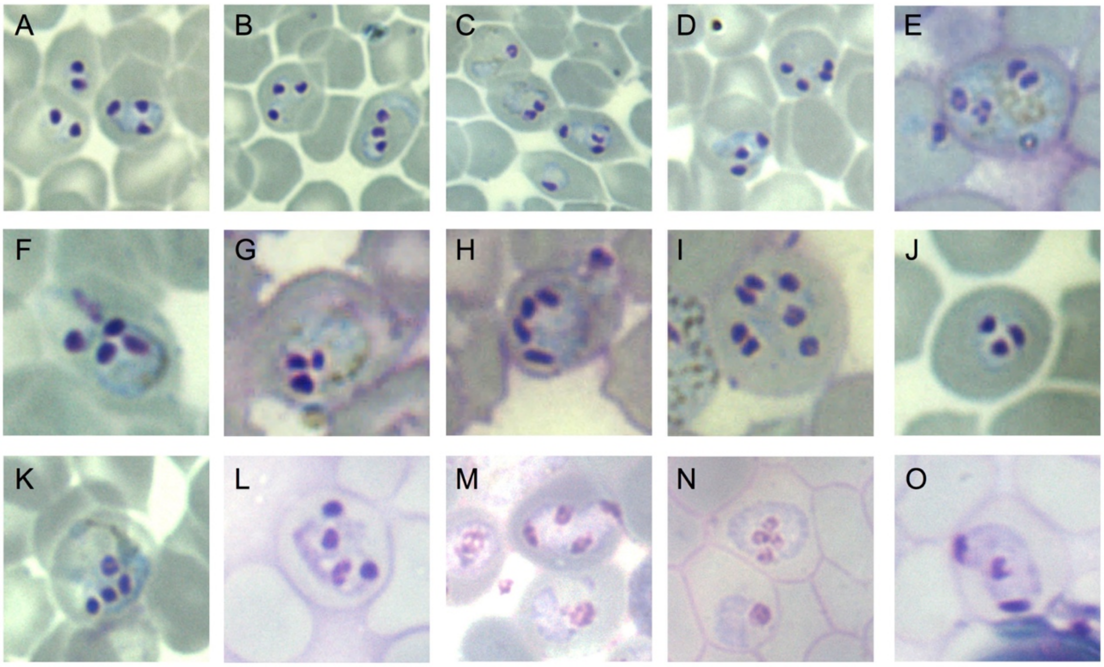

Supplemental Figure S2. Representative micrographs depicting blood stages of *Hepatocystis* parasites of *Epomophorus* hosts. Three individuals of *Epomophorus* sp. (DMR630, DMR631, DMR634), sampled during the wet season, featured blood stages with an unusual morphology. (A) early blood stages are annular or ovoid shaped with a peripheral nucleus. (B-E) separation of the nucleus in two or three parts that seem to be isolated from each other, but connected by the parasite´s cytoplasm. (C,D,K,L) Mid-adult stages with amoeboid shape. (I) parasite cell forms with nuclei, which could potentially be in the process of division (A, E-O) cytoplasm of the parasites stains pale blue and contains one or more chromatic masses that can be present in dense forms, in small compact blocks, rounded or elongated, arranged in linear or irregular clusters.

It was noted that during the preparation of the blood smear microgametocytes can undergo a rapid development during the time interval of fixation and drying (Garnham, 1966). A delay in drying the material might lead to the onset of the nuclear divisions in the microgametocyte. All samples of the current study were prepared in the same manner, but we cannot entirely exclude ambient humidity or a slight delay in preparation might have created conditions suitable for the process of exflagellation to start (in DMR630, DMR631, DMR634).

Reference

Garnham, P.C.C. Malaria parasites and other Haemosporidia. (Blackwell Scientific Publications, 1966).

Schaer *et al.*, Supplemental Table S1

Table S1: prevalence of *Hepatocystis* infections

**Bat suborder**	**Bat family**	**Host genus**[a]	**Prevalence**
Yinpterochiroptera	Hipposideridae	***Hipposideros***	**4 (1/23)**
	Pteropodidae	***Epomophorus***	**94 (130/138)**
		Epomops	**90 (9/10)**
		Hypsignathus	**100 (1/1)**
		Micropteropus	**89 (31/35)**
		Rousettus	0 (0/2)
	Rhinolophidae	*Rhinolophus**	0 (0/18)
Yangochiroptera	Emballonuridae	*Coleura afra*	0 (0/2)
		Taphozous	0 (0/2)
	Miniopteridae	*Miniopterus*	0 (0/2)
	Molossidae	*Molossidae* indet.[b]	0 (0/69)
	Nycteridae	*Nycteris**	0 (0/23)
	Vespertilionidae	*Glauconycteris*	0 (0/15)
		Neoromicia	0 (0/7)
		Niumbaha	0 (0/2)
		Pipistrellus	0 (0/1)
		Scotoecus	0 (0/15)
		Scotophilus	0 (0/28)
All investigated bats			**43.8 (172/393)**
Prevalence among infected genera			**83.1 (172/207)**
Prevalence among infected genera (excluding *Hipposideros*)			**92.9 (171/184)**

[a]Infected species are highlighted in bold and prevalences are given with numbers of infected per captured individuals. [b]Bats were only determined to the family level and most likely contain species of *Chaerephon*. *some individuals were host to the haemosporidian genus *Nycteria* (published in Schaer et al., 2015).

Schaer *et al.*, Supplemental Table S2

Supplemental Table S2: Screening results and parasitemia

Sample No.	USNM No.	Bat species	Blood smear	PCRs	Parasitemia in %
**DMR 111**	**590824**	***Micropteropus pusillus***	**positive**	**n.t.**	**0.085**
DMR 115	590828	*Rousettus aegyptiacus*	negative	negative	---
**DMR 116**	**590829**	***Micropteropus pusillus***	**negative**	**positive**	**< 0.001**
**DMR 118**	**590831**	***Epomophorus* sp.**	**positive**	**n.t.**	**< 0.001**
**DMR 119**	**590832**	***Epomophorus* sp.**	**negative**	**positive**	**< 0.001**
**DMR 120**	**590833**	***Micropteropus pusillus***	**positive**	**n.t.**	**0.015**
**DMR 123**	**590836**	***Micropteropus pusillus***	**negative**	**positive**	**< 0.001**
**DMR 125**	**590838**	***Micropteropus pusillus***	**negative**	**positive**	**< 0.001**
**DMR 126**	**590839**	***Epomophorus* sp.**	**negative**	**positive**	**< 0.001**
**DMR 131**	**590844**	***Micropteropus pusillus***	**positive**	**n.t.**	**0.049**
**DMR 132**	**590845**	***Micropteropus pusillus***	**negative**	**positive**	**< 0.001**
**DMR 133**	**590826**	***Epomophorus* sp.**	**positive**	**n.t.**	**< 0.001**
**DMR 134**	**590846**	***Micropteropus pusillus***	**positive**	**positive**	**0.038**
**DMR 135**	**590940**	***Micropteropus pusillus***	**negative**	**positive**	**< 0.001**
**DMR 136**	**590847**	***Epomophorus* sp.**	**positive**	**n.t.**	**< 0.001**
**DMR 137**	**590848**	***Epomophorus* sp.**	**negative**	**positive**	**< 0.001**
**DMR 139**	**590850**	***Epomophorus* sp.**	**negative**	**positive**	**< 0.001**
**DMR 160**	**590871**	***Epomophorus* sp.**	**positive**	**n.t.**	**0.014**
**DMR 161**	**590872**	***Epomophorus* sp.**	**positive**	**positive**	**0.087**
**DMR 162**	**590873**	***Epomophorus* sp.**	**positive**	**positive**	**1.406**
**DMR 163**	**590874**	***Epomophorus* sp.**	**positive**	**n.t.**	**1.136**
**DMR 164**	**590875**	***Epomophorus* sp.**	**positive**	**n.t.**	**< 0.001**
**DMR 165**	**590876**	***Epomophorus* sp.**	**positive**	**n.t.**	**0.123**
**DMR 166**	**590877**	***Epomophorus* sp.**	**positive**	**n.t.**	**0.019**
**DMR 167**	**590878**	***Epomophorus* sp.**	**positive**	**n.t.**	*****
**DMR 174**	**590885**	***Micropteropus pusillus***	**positive**	**n.t.**	**0.198**
**DMR 175**	**590866**	***Epomophorus* cf. *labiatus***	**positive**	**positive**	**2.955**
**DMR 176**	**590887**	***Micropteropus pusillus***	**negative**	**positive**	**< 0.001**
**DMR 177**	**590888**	***Epomophorus* cf. *labiatus***	**positive**	**n.t.**	**1.023**
**DMR 178**	**590889**	***Micropteropus pusillus***	**positive**	**positive**	**0.094**
**DMR 186**	**590897**	***Epomophorus* sp.**	**positive**	**n.t.**	**0.018**
**DMR 193**	**590904**	***Micropteropus pusillus***	**positive**	**n.t.**	**0.368**
DMR 195	590906	*Micropteropus pusillus*	negative	negative	---
**DMR 217**	**590928**	***Epomophorus* sp.**	**positive**	**n.t.**	**0.016**
**DMR 218**	**590929**	***Epomophorus* sp.**	**negative**	**positive**	**< 0.001**
**DMR 219**	**590930**	***Epomophorus* sp.**	**positive**	**positive**	**0.563**
**DMR 220**	**590931**	***Epomophorus* sp.**	**positive**	**n.t.**	**0.072**
**DMR 221**	**590932**	***Epomophorus* sp.**	**positive**	**n.t.**	**0.047**
**DMR 223**	**590934**	***Epomophorus* sp.**	**positive**	**n.t.**	**< 0.001**
**DMR 224**	**590935**	***Epomophorus* sp.**	**positive**	**n.t.**	**0.134**
**DMR 225**	**590936**	***Epomophorus* sp.**	**positive**	**n.t.**	**0.081**
**DMR 226**	**590937**	***Epomophorus* sp.**	**positive**	**n.t.**	**0.051**
**DMR 227**	**590938**	***Epomophorus* sp.**	**too bad**	**positive**	*****
DMR 255	586921	*Hipposideros* cf. *ruber*	negative	negative	---
DMR 259	586925	*Hipposideros* sp.	negative	negative	---
DMR 261	586927	*Hipposideros* sp.	negative	negative	---
DMR 263	586929	*Hipposideros* cf. *ruber*	negative	negative	---
DMR 264	586930	*Hipposideros* cf. *ruber*	negative	negative	---
DMR 266	586932	*Hipposideros* cf. *ruber*	negative	negative	---
DMR 267	586933	*Hipposideros* sp.	negative	negative	---
DMR 268	586934	*Hipposideros* sp.	negative	negative	---
DMR 272	586938	*Micropteropus pusillus*	negative	negative	---
**DMR 285**	**586951**	***Micropteropus pusillus***	**positive**	**positive**	**< 0.001**
**DMR 299**	**586965**	***Epomophorus* sp.**	**positive**	**positive**	**0.734**
DMR 338	587004	*Hipposideros* sp.	negative	negative	---
DMR 339	587005	*Hipposideros* sp.	negative	negative	---
DMR 341	587007	*Hipposideros* sp.	negative	negative	---
**DMR 342**	**587008**	***Hipposideros* sp.**	**positive**	**positive**	**< 0.001**
DMR 343	587009	*Hipposideros* sp.	negative	negative	---
DMR 344	587010	*Hipposideros* cf. *ruber*	negative	negative	---
DMR 345	587011	*Hipposideros* sp.	negative	negative	---
**DMR 350**	**587016**	***Micropteropus pusillus***	**positive**	**n.t.**	**< 0.001**
**DMR 352**	**587018**	***Epomophorus* sp.**	**negative**	**positive**	**< 0.001**
**DMR 354**	**587020**	***Epomophorus* sp.**	**negative**	**positive**	**< 0.001**

**DMR 355**	**587021**	***Epomophorus* sp.**	**positive**	**n.t.**	**0.124**
**DMR 358**	**587024**	***Epomophorus* sp.**	**positive**	**n.t.**	**0.126**
**DMR 360**	**587025**	***Epomophorus* sp.**	**positive**	**positive**	**3.472**
**DMR 364**	**587029**	***Epomophorus* sp.**	**positive**	**n.t.**	**0.922**
**DMR 367**	**587032**	***Micropteropus pusillus***	**positive**	**positive**	**0.069**
**DMR 368**	**587033**	***Epomophorus* sp.**	**negative**	**positive**	**< 0.001**
**DMR 369**	**587034**	***Epomophorus* sp.**	*****	**positive**	*****
**DMR 372**	**587037**	***Epomophorus* sp.**	**positive**	**n.t.**	**0.485**
**DMR 373**	**587038**	***Epomophorus* sp.**	**positive**	**positive**	**0.949**
**DMR 374**	**587039**	***Epomophorus* sp.**	**positive**	**n.t.**	**0.392**
DMR 375	587040	*Epomophorus* sp.	negative	negative	---
**DMR 376**	**587041**	***Epomophorus* sp.**	**positive**	**n.t.**	**0.080**
**DMR 396**	**587060**	***Epomophorus* sp.**	**positive**	**n.t.**	**0.073**
DMR 398	587062	*Micropteropus pusillus*	negative	negative	---
**DMR 399**	**587063**	***Epomophorus* sp.**	**positive**	**n.t.**	**< 0.001**
**DMR 414**	**587078**	***Epomophorus* sp.**	**positive**	**positive**	**2.397**
**DMR 415**	**587079**	***Epomophorus* sp.**	**positive**	**n.t.**	**< 0.001**
**DMR 416**	**587080**	***Epomophorus* sp.**	**negative**	**positive**	**< 0.001**
**DMR 417**	**587081**	***Epomophorus* sp.**	**positive**	**n.t.**	**1.525**
**DMR 418**	**587082**	***Epomophorus* sp.**	**positive**	**n.t.**	**0.112**
**DMR 419**	**587083**	***Epomophorus* sp.**	**positive**	**n.t.**	**0.397**
**DMR 464**	**587126**	***Epomophorus* sp.**	**positive**	**n.t.**	**0.019**
**DMR 465**	**587127**	***Epomophorus* sp.**	**positive**	**n.t.**	**0.081**
**DMR 466**	**587128**	***Epomophorus* sp.**	**positive**	**n.t.**	**0.751**
**DMR 467**	**587129**	***Epomophorus* sp.**	**positive**	**n.t.**	**0.175**
**DMR 468**	**587130**	***Epomophorus* sp.**	**positive**	**n.t.**	**0.209**
**DMR 469**	**587131**	***Epomophorus* sp.**	**positive**	**n.t.**	**0.016**
**DMR 473**	**587135**	***Epomophorus* sp.**	**positive**	**n.t.**	**0.021**
**DMR 474**	**587136**	***Epomophorus* sp.**	**positive**	**n.t.**	**0.483**
**DMR 475**	**587137**	***Epomophorus* sp.**	**positive**	**positive**	**0.082**
**DMR 476**	**587138**	***Epomophorus* sp.**	**positive**	**n.t.**	**0.065**
**DMR 477**	**587139**	***Micropteropus pusillus***	**positive**	**n.t.**	**0.025**
**DMR 478**	**587140**	***Epomophorus* sp.**	**positive**	**n.t.**	**0.204**
**DMR 480**	**587142**	***Epomophorus* sp.**	**positive**	**n.t.**	**< 0.001**
DMR 481	587143	*Epomophorus* sp.	negative	negative	---
**DMR 482**	**587144**	***Epomophorus* sp.**	**positive**	**n.t.**	**0.382**
DMR 483	587145	*Epomophorus* sp.	negative	negative	---
**DMR 484**	**587146**	***Epomophorus* sp.**	**positive**	**n.t.**	**< 0.001**
**DMR 485**	**587147**	***Epomophorus* sp.**	**positive**	**n.t.**	**0.014**
**DMR 486**	**587148**	***Epomophorus* sp.**	**positive**	**n.t.**	**< 0.001**
**DMR 487**	**587149**	***Epomophorus* sp.**	**positive**	**positive**	**0.973**
**DMR 488**	**587150**	***Epomophorus* sp.**	**positive**	**n.t.**	**0.050**
**DMR 489**	**587151**	***Epomophorus* sp.**	**positive**	**n.t.**	**0.226**
**DMR 490**	**587152**	***Epomophorus* sp.**	**positive**	**n.t.**	**0.034**
DMR 499	587161	*Micropteropus pusillus*	negative	negative	---
**DMR 543**	**587204**	***Epomophorus* sp.**	**positive**	**positive**	**< 0.001**
DMR 545	587206	*Epomophorus* sp.	negative	negative	---
**DMR 546**	**587207**	***Micropteropus pusillus***	**positive**	**positive**	**4.657**
**DMR 548**	**587209**	***Micropteropus pusillus***	**positive**	**n.t.**	**0.015**
**DMR 553**	**587214**	***Hypsignathus monstrosus***	**positive**	**positive**	**0.503**
**DMR 558**	**587219**	***Micropteropus pusillus***	**positive**	**positive**	**1.959**
**DMR 570**	**587225**	***Micropteropus pusillus***	**positive**	**n.t.**	**0.112**
**DMR 571**	**587226**	***Micropteropus pusillus***	**positive**	**n.t.**	**0.840**
DMR 574	587229	*Hipposideros gigas*	negative	negative	---
**DMR 575**	**587230**	***Epomops franqueti***	**positive**	**n.t.**	**0.193**
**DMR 577**	**587232**	***Epomops franqueti***	**positive**	**n.t.**	**0.201**
**DMR 578**	**587233**	***Epomops franqueti***	**positive**	**n.t.**	**0.010**
**DMR 579**	**587234**	***Epomops franqueti***	**negative**	**positive**	**< 0.001**
DMR 582	587237	*Rousettus aegyptiacus*	negative	negative	---
DMR 584	587239	*Epomops franqueti*	negative	negative	---
**DMR 592**	**587247**	***Epomops franqueti***	**positive**	**positive**	**0.339**
DMR 593	587248	*Hipposideros abae*	negative	negative	---
**DMR 594**	**587249**	***Epomops franqueti***	**positive**	**n.t.**	**0.032**
**DMR 595**	**587250**	***Epomops franqueti***	**positive**	**positive**	**0.186**
**DMR 596**	**587251**	***Epomops franqueti***	**positive**	**positive**	**< 0.001**
DMR 597	587252	*Hipposideros abae*	negative	negative	---
DMR 598	587253	*Hipposideros abae*	negative	negative	---
**DMR 599**	**587254**	***Epomops franqueti***	**positive**	**positive**	**0.175**
**DMR 603**	**587257**	***Micropteropus pusillus***	**positive**	**n.t.**	**0.219**
**DMR 604**	**587258**	***Micropteropus pusillus***	**positive**	**n.t.**	**0.298**
**DMR 605**	**587259**	***Micropteropus pusillus***	**positive**	**positive**	**0.157**

**DMR 608**	**587261**	***Micropteropus pusillus***	**positive**	**positive**	**0.129**
DMR 626	587279	*Hipposideros* sp.	negative	negative	---
DMR 627	587280	*Hipposideros abae*	negative	negative	---
**DMR 628**	**587281**	***Epomophorus* sp.**	**positive**	**n.t.**	**0.972**
**DMR 629**	**587282**	***Epomophorus* sp.**	**positive**	**n.t.**	**0.040**
**DMR 630**	**587283**	***Epomophorus* sp.**	**positive**	**positive**	**2.759**
**DMR 631**	**587284**	***Epomophorus* sp.**	**positive**	**positive**	**0.241**
**DMR 632**	**587285**	***Epomophorus* sp.**	**positive**	**positive**	**2.121**
**DMR 633**	**587286**	***Epomophorus* sp.**	**positive**	**positive**	**0.064**
**DMR 634**	**587287**	***Epomophorus* sp.**	**positive**	**positive**	**2.994**
**DMR 635**	**587288**	***Epomophorus* sp.**	**positive**	**n.t.**	**0.287**
**DMR 637**	**587290**	***Epomophorus* sp.**	**positive**	**n.t.**	**0.229**
**DMR 638**	**587291**	***Epomophorus* sp.**	**positive**	**n.t.**	**4.440**
**DMR 639**	**587292**	***Epomophorus* sp.**	**positive**	**n.t.**	**2.529**
**DMR 640**	**587293**	***Epomophorus* sp.**	**positive**	**n.t.**	**837**
**DMR 641**	**587294**	***Epomophorus* sp.**	**positive**	**n.t.**	**0.435**
**DMR 642**	**587295**	***Epomophorus* sp.**	**positive**	**n.t.**	**0.378**
**DMR 643**	**587296**	***Epomophorus* sp.**	**positive**	**n.t.**	**0.198**
**DMR 644**	**587297**	***Epomophorus* sp.**	**positive**	**n.t.**	**0.105**
**DMR 645**	**587298**	***Epomophorus* sp.**	**positive**	**n.t.**	**0.395**
**DMR 646**	**587299**	***Epomophorus* sp.**	**positive**	**n.t.**	**2.400**
DMR 687	586773	*Hipposideros* cf. *ruber*	negative	negative	---
**DMR 702**	**586788**	***Epomophorus* sp.**	**positive**	**n.t.**	**0.025**
**DMR 703**	**586789**	***Micropteropus pusillus***	**positive**	**n.t.**	**0.415**
**DMR 704**	**586790**	***Micropteropus pusillus***	**positive**	**positive**	**0.337**
**DMR 706**	**586792**	***Micropteropus pusillus***	**positive**	**n.t.**	**0.042**
**DMR 715**	**586801**	***Epomophorus* sp.**	**positive**	**n.t.**	**0.087**
DMR 717	586803	*Hipposideros* sp.	negative	negative	---
**DMR 727**	**586813**	***Epomophorus* sp.**	**positive**	**positive**	**0.215**
**DMR 728**	**586814**	***Epomophorus* sp.**	**positive**	**n.t.**	**0.035**
**DMR 729**	**586815**	***Micropteropus pusillus***	**negative**	**positive**	**< 0.001**
**DMR 731**	**586817**	***Micropteropus pusillus***	**negative**	**positive**	**< 0.001**
**DMR 733**	**586819**	***Epomophorus* sp.**	**positive**	**positive**	**0.105**
**DMR 734**	**586820**	***Epomophorus gambianus***	**positive**	**n.t.**	**0.074**
**DMR 735**	**586821**	***Epomophorus gambianus***	**positive**	**positive**	**0.171**
**DMR 737**	**586823**	***Epomophorus* sp.**	**positive**	**n.t.**	**0.088**
**DMR 738**	**586824**	***Epomophorus* sp.**	**positive**	**positive**	**0.726**
**DMR 783**	**587632**	***Epomophorus* sp.**	**positive**	**positive**	**0.067**
DMR 784	587633	*Epomophorus* sp.	negative	negative	---
**DMR 788**	**587636**	***Epomophorus* sp.**	**positive**	**n.t.**	**< 0.001**
**DMR 790**	**587638**	***Epomophorus* sp.**	**positive**	**n.t.**	**0.030**
**DMR 791**	**587639**	***Epomophorus* sp.**	**negative**	**positive**	**< 0.001**
**DMR 792**	**587640**	***Epomophorus* sp.**	**positive**	**positive**	**0.103**
**DMR 793**	**587641**	***Epomophorus* sp.**	**positive**	**n.t.**	**0.013**
**DMR 794**	**587642**	***Epomophorus* sp.**	**positive**	**n.t.**	**0.054**
**DMR 842**	**587690**	***Epomophorus* sp.**	**positive**	**positive**	**< 0.001**
**DMR 843**	**587691**	***Epomophorus* sp.**	**negative**	**positive**	**< 0.001**
**DMR 844**	**587692**	***Epomophorus* sp.**	**positive**	**n.t.**	**< 0.001**
**DMR 845**	**587693**	***Epomophorus* sp.**	**positive**	**positive**	**0.075**
**DMR 846**	**587694**	***Epomophorus* sp.**	**positive**	**positive**	**0.053**
**DMR 847**	**587695**	***Epomophorus* sp.**	**negative**	**positive**	**< 0.001**
**DMR 848**	**587696**	***Epomophorus* sp.**	**positive**	**positive**	**0.029**
**DMR 849**	**587697**	***Epomophorus* sp.**	**positive**	**n.t.**	**0.041**
**DMR 850**	**587698**	***Epomophorus* sp.**	**positive**	**n.t.**	**< 0.001**
**DMR 851**	**587699**	***Epomophorus* sp.**	**positive**	**n.t.**	**0.020**
**DMR 852**	**587700**	***Epomophorus* sp.**	**positive**	**n.t.**	**< 0.001**
**DMR 853**	**587701**	***Epomophorus* sp.**	**positive**	**n.t.**	**0.068**
**DMR 855**	**587703**	***Epomophorus* sp.**	**positive**	**n.t.**	**0.038**
**DMR 857**	**587705**	***Epomophorus* sp.**	**positive**	**positive**	**0.027**
**DMR 862**	**587710**	***Epomophorus* sp.**	**positive**	**positive**	**< 0.001**
**DMR 864**	**587712**	***Epomophorus* sp.**	**positive**	**n.t.**	**0.030**
**DMR 868**	**587716**	***Epomophorus* sp.**	**negative**	**positive**	**< 0.001**
**DMR 869**	**587717**	***Epomophorus* sp.**	**negative**	**positive**	**< 0.001**
**DMR 870**	**587718**	***Epomophorus* sp.**	**negative**	**positive**	**< 0.001**
**DMR 871**	**587719**	***Epomophorus* sp.**	**negative**	**positive**	**< 0.001**
DMR 872	587720	*Epomophorus* sp.	negative	negative	---
DMR 873	587721	*Epomophorus* sp.	negative	negative	---
**DMR 884**	**587732**	***Epomophorus* sp.**	**positive**	**n.t.**	**0.207**
**DMR 891**	**587739**	***Epomophorus* sp.**	**positive**	**n.t.**	**0.032**
**DMR 892**	**587740**	***Epomophorus* sp.**	**positive**	**positive**	**0.042**
DMR 893	587741	*Epomophorus* sp.	negative	negative	---

**DMR 894**	**587742**	***Epomophorus* sp.**	**positive**	**positive**	**0.088**
**DMR 900**	**587748**	***Epomophorus* sp.**	**positive**	**positive**	**0.154**
**DMR 901**	**587749**	***Epomophorus* sp.**	**positive**	**n.t.**	**0.077**
**K895****		***Epomophorus labiatus***	**positive**	**positive**	*****
**K966****		***Epomophorus wahlbergi***	**positive**	**positive**	*****

Table S2 lists all investigated individuals of the bat host families Pteropodidae and Hipposideridae. Positive samples are highlighted in bold. Results of microscopical screening and PCR detection methods are given as well as parasitemia values in %. *blood smear quality not sufficient for determination of parasitemia; n.t. = not tested by PCR; **collected in Kenya 2009

Schaer *et al*., Supplemental Table S3

Supplemental Table S3. GenBank accession numbers with samples from this study highlighted in bold.

				Parasite sequence			
**Parasite group**	**Sample**	**Host group**	**locality**	**Cytb**	**Cox1**	**Clpc**	**Ef2**
Polychromophilus	*Polychromophilus melanipherus*	*Miniopterus schreibersii*	Switzerland	JN990709	JN990715	JN990721	—
	Polychromophilus murinus	*Myotis daubentonii*	Switzerland	HM055583	JN990718	JN990723	—
	Polychromophilus sp (M villiersi)	*Miniopterus villiersi*	Guinea	KF159699	KF159795	KF159616	KF159740
	Polychromophilus sp (Pip grandidieri)	*Pipistrellus grandidieri*	Guinea	KF159714	KF159797	KF159639	KF159742
Nycteria	*Nycteria sp (Rh landeri)*	*Rhinolophus landeri*	Guinea	KF159690	KF159787	—	—
	Nycteria (Rhino DMR257)	*Rhinolophus* sp	South Sudan	KP053768	—	KP053794	KP053775
	Nycteria (R hildebrandti 967)	*Rhinolophus hildebrandti*	Kenya	KP053763	—	KP053788	KP053770
Rodent *Plasmodium*	*Plasmodium berghei (ANKA)*	*Grammomys surdaster*[1]	DR Congo	DQ414645	DQ414589	DQ417612	—
	Plasmodium chabaudi (AS)	*T. rutilans*[2]	CAR	DQ414649	DQ414593	DQ417616	XM_736543
	Plasmodium vinckei	*Grammomys surdaster*[1]	DR Congo	DQ414651	DQ414596	DQ417619	—
	Plasmodium yoelii	*T. rutilans*[2]	CAR	AY099051	DQ414605	DQ417628	LM993667
Bat *Plasmodium*	*Plasmodium cyclopsi*	*Hipposideros cyclops*	Liberia	KF159710	KF159788	KF159635	KF159729
	Plasmodium voltaicum	*Myonycteris angolensis*	Guinea	KF159671	KF159792	KF159648	—
Primate *Plasmodium*	*Plasmodium cynomolgi*	Old World monkeys	Ceylon	AF069616	AB444126	AB471873	XM_004223974
	Plasmodium falciparum	Humans	Honduras	DQ642845	M76611	DQ642846	DQ642846
	Plasmodium gaboni	*Pan troglodytes*	Gabon	FJ895307	FJ895307	HQ842630	XM_018788194
	Plasmodium knowlesi	Old World monkeys	Malaysia	AF069621	AY598141	AF348341	XM_002260326
	Plasmodium malariae	Humans	Uganda	AF069624	AB489193	AF348342	—
	Plasmodium reichenowi	*Pan troglodytes*	Africa	AJ251941	AJ251941	EU560464	—
	Plasmodium sp (Chimp_C1)	*Pan* sp	DR Congo	HM235391	HM235391	HM235147	—
	Plasmodium sp (Gorilla_G1)	*Gorilla* sp	Cameroon	HM235288	HM235308	HM235163	—
	Plasmodium ovale	Humans	Bangladesh	AF069625	JF894415	AY634623	—
	Plasmodium vivax	Humans	Brazil	AF069619	AAY26841	AF348344	XM_001615828
Primate *Hepatocystis*	*Cerco_cephus_Gabon*	*Cercopithecus cephus*	Gabon	JF923760	—	—	—
	Cerco_cephus_Gabon	*Cercopithecus cephus*	Gabon	JF923758	—	—	—
	Cerco_nictitans_Cameroon	*Cercopithecus nictitans*	Cameroon	JQ070956	—	—	—
	Cerco_nictitans_Cameroon	*Cercopithecus nictitans*	Cameroon	JQ070816	—	—	—
	Cerco_nictitans_Cameroon	*Cercopithecus nictitans*	Cameroon	JQ070814	—	—	—
	Cerco_nictitans_Cameroon	*Cercopithecus nictitans*	Cameroon	JQ070955	—	—	—
	Macaque_Thailand	*Macaca* sp	Thailand	EU400409	—	—	—
	Macaque_Southeast Asia	*Macaca* sp	Southeast Asia	GU929945	—	—	—
	Macaque_Southeast Asia	*Macaca* sp	Southeast Asia	GU930036	—	—	—
	Macaque_Southeast Asia	*Macaca* sp	Southeast Asia	GU929944	—	—	—
	Macaque_Myanmar	*Macaca* sp	Myanmar	HQ605040	—	—	—
	Mandrillus_sphinx_Gabon	*Mandrillus sphinx*	Gabon	JF923759	—	—	—

	Miopithecus_talapoin_Gabon	*Miopithecus talapoin*	Gabon	JF923757	—	—	—
	monkey_Kibale_Uganda	monkey	Uganda	KC262835	—	—	—
	monkey_Kibale_Uganda	monkey	Uganda	KC262799	—	—	—
	monkey_Kibale_Uganda	monkey	Uganda	KC262802	—	—	—
	monkey_Kibale_Uganda	monkey	Uganda	KC262797	—	—	—
	monkey_Kibale_Uganda	monkey	Uganda	KC262824	—	—	—
	African monkey_Kibale_Uganda	monkey	Uganda	KC262867	—	—	—
	Papio nubensis_Ethiopia	*Papio nubensis*	Ethiopia	AF069626	—	—	—
	Procolo_badius_Kibale Uganda	*Procolobus badius*	Uganda	GU945305	—	—	—
	Procolo_badius_Kibale Uganda	*Procolobus badius*	Uganda	GU945285	—	—	—
	Procolo_badius_Kibale Uganda	*Procolobus badius*	Uganda	GU945284	—	—	—
Bat *Hepatocystis*	*Cyn_brach_Sin1*	*Cynopterus brachyotis*	Singapore	EU254526	EU254569	EU254616	—
	Epom_Guinea_G4_1	*Epomophorus gambianus*	Guinea	KF159695	—	—	—
	Epo_SoSu_WE_DMR592	***Epomops franqueti***	**South Sudan (WS)**	**KY753503**	**KY753528**	**KY753544**	**KY753568**
	Epo_SoSu_WS_DMR595	***Epomops franqueti***	**South Sudan (WS)**	**KY753504**	—	**KY753545**	**KY753569**
	Epom_Kenya_K895	***Epomophorus labiatus***	**Kenya**	**KY753518**	**KY753536**	**KY753559**	**KY753583**
	Epom_Kenya_K966	***Epomophorus wahlbergi***	**Kenya**	**KY753519**	—	**KY753560**	**KY753584**
	Epom_SoSu_CS_DMR161	***Epomophorus* sp**	**South Sudan (CS)**	**KY753505**	**KY753529**	**KY753546**	**KY753570**
	Epom_SoSu_CS_DMR162	***Epomophorus* sp**	**South Sudan (CS)**	**KY753506**	**KY753530**	**KY753547**	**KY753571**
	Epom_SoSu_CS_DMR299	***Epomophorus* sp**	**South Sudan (CS)**	**KY753507**	**KY753531**	**KY753548**	**KY753572**
	Epom_SoSu_CS_DMR360	***Epomophorus* sp**	**South Sudan (CS)**	**KY753508**	**KY753532**	**KY753549**	**KY753573**
	Epom_SoSu_CS_DMR738	***Epomophorus* sp**	**South Sudan (CS)**	**KY753514**	—	**KY753555**	**KY753579**
	Epom_SoSu_CS_DMR845	***Epomophorus* sp**	**South Sudan (CS)**	**KY753515**	—	**KY753556**	**KY753580**
	Epom_SoSu_CS_DMR862	***Epomophorus* sp**	**South Sudan (CS)**	**KY753516**	—	**KY753557**	**KY753581**
	Epom_SoSu_CS_DMR892	***Epomophorus* sp**	**South Sudan (CS)**	**KY753517**	—	**KY753558**	**KY753582**
	Epom_SoSu_WS_DMR543	***Epomophorus* sp**	**South Sudan (WS)**	**KY753509**	**KY753533**	**KY753550**	**KY753574**
	Epom_SoSu_WS_DMR634	***Epomophorus* sp**	**South Sudan (WS)**	**KY753510**	**KY753534**	**KY753551**	**KY753575**
	Epom_SoSu_WS_DMR727	***Epomophorus* sp**	**South Sudan (WS)**	**KY753511**	—	**KY753552**	**KY753576**
	Epom_SoSu_WS_DMR733	***Epomophorus* sp**	**South Sudan (WS)**	**KY753512**	—	**KY753553**	**KY753577**
	Epom_SoSu_WS_DMR735	***Epomophorus* sp**	**South Sudan (WS)**	**KY753513**	**KY753535**	**KY753554**	**KY753578**
	E_buett_Guinea_G4_1	*Epomops buettikoferi*	Guinea	KF159701	KF159768	KF159636	—
	E_buett_Guinea_G4_3	*Epomops buettikoferi*	Guinea	KF159703	KF159790	—	—
	E_buett_Guinea_G4_2	*Epomops buettikoferi*	Guinea	KF159706	KF159779	KF159612	KF159757
	E_buett_Liberia_L4_1	*Epomops buettikoferi*	Liberia	—	—	—	KF159741
	Epo_UGA25	*Epomops franqueti*	Uganda	KT750356	KT750527	KT750667	—
	Epo_UGA13	*Epomops franqueti*	Uganda	KT750351	KT750526	KT750668	—
	Epo_UGA07	*Epomops franqueti*	Uganda	KT750353	KT750524	KT750670	—
	Hipposideros_sp_DMR342	***Hipposideros* sp.**	**South Sudan (CS)**	**KY753520**	—	—	—
	Hipposideros_larvatus_Cambodia	*Hipposideros larvatus*	Cambodia	EF179356	—	—	—
	Hyps_Liberia_L4_1	*Hypsignathus monstrosus*	Liberia	KF159712	—	KF159647	—
	Hyps_Liberia_L2_1	*Hypsignathus monstrosus*	Liberia	KF159689	KF159799	—	KF159734
	Hyps_SoSu_WS_DMR553	***Hypsignathus monstrosus***	**South Sudan (WS)**	**KY753521**	**KY753537**	**KY753561**	**KY753585**

	Mic_Guinea_G4_5	*Micropteropus pusillus*	Guinea	KF159704	KF159775	KF159618	KF159753
	Mic_Guinea_G5_4	*Micropteropus pusillus*	Guinea	KF159680	KF159778	KF159641	KF159723
	Mic_Guinea_G5_6	*Micropteropus pusillus*	Guinea	KF159687	KF159781	KF159649	KF159756
	Mic_Guinea_G4_2	*Micropteropus pusillus*	Guinea	KF159693	KF159773	KF159645	KF159746
	Mic_Guinea_G1_4	*Micropteropus pusillus*	Guinea	KF159713	KF159759	KF159622	KF159738
	Mic_Guinea_G1_10	*Micropteropus pusillus*	Guinea	KF159683	KF159801	KF159623	KF159744
	Mic_SoSu_CS_DMR285	***Micropteropus pusillus***	**South Sudan (CS)**	**KY753524**	**KY753540**	**KY753564**	**KY753588**
	Mic_SoSu_CS_DMR178	***Micropteropus pusillus***	**South Sudan (CS)**	**KY753523**	**KY753539**	**KY753563**	**KY753587**
	Mic_SoSu_CS_DMR367	***Micropteropus pusillus***	**South Sudan (CS)**	**KY753525**	**KY753541**	**KY753565**	**KY753589**
	Mic_SoSu_WS_DMR558	***Micropteropus pusillus***	**South Sudan (WS)**	**KY753527**	**KY753543**	**KY753567**	**KY753591**
	Mic_SoSu_WS_DMR546	***Micropteropus pusillus***	**South Sudan (WS)**	**KY753526**	**KY753542**	**KY753566**	**KY753590**
	Mic_SoSu_CS_DMR134	***Micropteropus pusillus***	**South Sudan (CS)**	**KY753522**	**KY753538**	**KY753562**	**KY753586**
	Myo_lep_Liberia_L2_1	*Myonycteris leptodon*	Liberia	KF159707	KF159783	—	KF159755
	Myo_lep_Liberia_L4_1	*Myonycteris leptodon*	Liberia	KF159678	KF159784	—	KF159750
	Myo_lep_Ivory_C7_1	*Myonycteris leptodon*	Ivory Coast	KF188066	KF188069	—	KF188071
	Myo_lep_Liberia_L3_1	*Myonycteris leptodon*	Liberia	KF159705	—	—	KF159705
	Myo_tor_UGA26	*Myonycteris torquata*	Uganda	KT750357	KT750521	KT750673	—
	Myo_tor_UGA27	*Myonycteris torquata*	Uganda	KT750342	KT750534	KT750659	—
	Myo_tor_UGA24	*Myonycteris torquata*	Uganda	KT750343	KT750506	KT750687	—
	Nan_Guinea_1	*Nanonycteris veldkampii*	Guinea	EU254528	EU254571	EU254618	—
	Nan_Guinea_2	*Nanonycteris veldkampii*	Guinea	EU254527	EU254570	EU254617	—
	Nan_Liberia_L1_1	*Nanonycteris veldkampii*	Liberia	KF159698	KF159786	KF159631	KF159749
	Pteropus_hypo_Mal	*Pteropus hypomelanus*	Malaysia	FJ168565	FJ168565	—	—

[1]*Grammomys surdaster = Grammomys dolichurus* [2]*Thamnomys rutilans = Grammomys poensis*

Schaer *et al.*, Supplemental Table S4

Supplemental Table S4. Nucleotide primers used in this study

Gene	Primer name	Sequence (5´- 3´)	Reference
**Cytb**	Hep-F3	CTTACCTTGGGGACAAATGAGTTATT	Schaer et al., 2013
	Hep-R3	CTCTAGCACCAAATGTCATTTTAAATTG	Schaer et al., 2013
	DW2	TAATGCCTAGACGTATTCCTGATTATCCAG	Perkins and Schall, 2002
	DW4	TGTTTGCTTGGGAGCTGTAATCATAATGTG	Perkins and Schall, 2002
	3932-F	GGGTTATGTATTACCTTGGGGTC	Perkins and Schall, 2002
	3932-R	GACCCCAAGGTAATACATAACCC	Perkins and Schall, 2002
**Cox1**	Cox1-F	CTATTTATGGTTTTCATTTTTATTTGGTA	Martinsen et al., 2008
	Cox1-R	AGGAATACGTCTAGGCATTACATTAAATCC	Martinsen et al., 2008
	Cox-in-F	ATGATATTTACARTTCAYGGWATTATTATG	Martinsen et al., 2008
	Cox-in-R	GTATTTTCTCGTAATGTTTTACCAAAGAA	Martinsen et al., 2008
	Cox-mid-F	TTATTCTGGTTTTTTGGTCATCCAG	Martinsen et al., 2008
	Cox-mid-R	CTGGATGACCAAAAAACCAGAATAA	Martinsen et al., 2008
**Clpc**	Clpc-out-F	AAACTGAATTAGCAAAAATATTA	Martinsen et al., 2008
	Clpc-out-R	CGWGCWCCATATAAAGGAT	Martinsen et al., 2008
	Clpc-in-F	GATTTGATATGAGTGAATATATGG	Martinsen et al., 2008
	Clpc-in-R	CCATATAAAGGATTATAWG	Martinsen et al., 2008
**EF2**	EF2-F	GTTCGTGAGATCATGAACAAAAC	Schaer et al., 2013
	EF2-R	CCTTGTAAACCAGAACCAAA	Schaer et al., 2013

Cytb, cytochrome *b*; Cox1, cytochrome oxidase I; Clpc, apicoplast caseinolytic protease; EF2, nuclear elongation factor 2.

References

Martinsen, E.S., Perkins, S.L., Schall, J., 2008. A three-genome phylogeny of malaria parasites (*Plasmodium* and closely related genera): evolution of life-history traits and host switches. Mol. Phylogenet. Evol. 47, 261–273.

Perkins, S.L., Schall, J., 2002. A molecular phylogeny of malarial parasites recovered from cytochrome b gene sequences. J. Parasitol. 88, 972–978.

Schaer, J., Perkins, S.L., Decher, J., Leendertz, F.H., Fahr, J., Weber, N., Matuschewski, K., 2013. High diversity of West African bat malaria parasites and a tight link with rodent *Plasmodium* taxa. Proc. Natl. Acad. Sci. USA. 110. 17415- 7419.

Schaer *et al*., Supplemental Table S5

Supplemental Table S5. Final aligned sequence (seq) length, variable and parsimony informative characters for the mitochondrial, apicoplast and nuclear DNA.

Gene (genome)	Aligned seq. length	# sequences/ sequences in concatenated alignment	Missing data (%)	Variable sites	PICs	AT content (%)
**Cytb (mitochondrial)**	906	100/100	14.1	821 (90.6%)	317 (35.0%)	73.3
**Cox1 (mitochondrial)**	951	63/100	42.4	951 (100%)	327 (34.4%)	69.7
**Clpc (apicoplast)**	564	66/100	39.8	564 (100%)	165 (29.3%)	82.9
**Ef2 (nuclear)**	567	53/100	49.9	567 (100%)	147 (25.9%)	66.6
**Total/ Concatenated**	**2988**	**100**	**34.7**	**2903 (97.2%)**	**956 (32.0%)**	**73.0**

Final aligned sequence length does not contain any gaps, ambiguities have been converted to Ns. The concatenated alignment comprised a total length of 2988bp (including 906bp of cytochrome b, 951bp of cytochrome oxidase 1,564bp of the apicoplast Clpc and 567bp of the nuclear ef2 gene). The proportion of missing data (including data converted to Ns) was 35% (due to unavailability of published cox1-, Clpc- and especially ef2-sequences for most of the primate *Hepatocystis* sequences). No section of the alignments was ambiguous. The proportion of variable sites was 97% and informative sites were 32%.

Schaer *et al.*, Supplemental Table S6

Supplemental Table S6. Partitioning and evolution models used for the concatenated analyses (tested with *PartitionFinder*)

	RaxML (BIC)			MrBayes (BIC)		
**Subset**	**Subset Partitions**	**Subset Sites**	**Best Model**	**Partition names**	**# sites**	**Best Model**
1	clpc_pos1, cox1_pos1, cytb_pos1	1-906\3, 907-1857\3, 1858-2421\3	GTR+I+G	clpc_pos1, clpc_pos2, cox1_pos1, cytb_pos1	1-906\3, 907-1857\3, 1858-2421\3, 1859-2421\3	GTR+I+G
2	clpc_pos2, cox1_pos2, cytb_pos2, ef2_pos2	2-906\3, 908-1857\3, 1859-2421\3, 2423-2988\3	GTR+I+G	cox1_pos2, cytb_pos2	2-906\3, 908-1857\3	GTR+I+G
3	cox1_pos3, cytb_pos3	3-906\3, 909-1857\3	GTR+G	cox1_pos3, cytb_pos3	3-906\3, 909-1857\3	GTR+G
4	clpc_pos3	1860-2421\3	GTR+G	clpc_pos3	1860-2421\3	GTR+G
5	ef2_pos1	2422-2988\3	GTR+I+G	ef2_pos1, ef2_pos2	2422-2988\3, 2423-2988\3	JC+I
6	ef2_pos3	2424-2988\3	GTR+G	ef2_pos3	2424-2988\3	HKY+G
